# Ruin Analysis on a New Risk Model with Stochastic Premiums and Dependence Based on Time Series for Count Random Variables

**DOI:** 10.3390/e25040698

**Published:** 2023-04-21

**Authors:** Lihong Guan, Xiaohong Wang

**Affiliations:** 1 School of Science, Changchun University, Changchun 130022, China; 2 Mathematics and Computer College, Jilin Normal University, Siping 136000, China

**Keywords:** risk model, stochastic premiums, INAR(1) process, INMA(1) process, ruin probability, 62P05, 91B30, 97M30

## Abstract

In this paper, we propose a new discrete-time risk model of an insurance portfolio with stochastic premiums, in which the temporal dependence among the premium numbers of consecutive periods is fitted by the first-order integer-valued autoregressive (INAR(1)) process and the temporal dependence among the claim numbers of consecutive periods is described by the integer-valued moving average (INMA(1)) process. To measure the risk of the model quantitatively, we study the explicit expression for a function whose solution is defined as the Lundberg adjustment coefficient and give the Lundberg approximation formula for the infinite-time ruin probability. In the case of heavy-tailed claim sizes, we establish the asymptotic formula for the finite-time ruin probability via the large deviations of the aggregate claims. Two numerical examples are provided in order to illustrate our theoretical findings.

## 1. Introduction

As an absolutely necessary part of the modern financial system, insurance is one of the most effective ways for people to manage risks, such that it plays a significant role in our daily life. A very important task of insurance companies is to quantitatively analyze future claims. Consequently, risk theory has become an active research field of actuarial science. For the classical mathematical risk model, the so-called Lundberg–Cramér surplus process has the following form:

(1) $$\begin{array}{c} U_{t}^{0}=u+ct-\sum_{i=1}^{N_{t}^{0}}Y_{i},\;\;t\geq 0, \end{array}$$

in which u≥0 is the initial capital of an insurance portfolio, c>0 is the constant rate of premium income, $\left\{N_{t}^{0},t\geq 0\right\}$ is a homogeneous Poisson process with intensity λ, the total claim numbers are denoted up to time *t*, and $Y_{i}$ describes the size of the *i*th claim. In the literature, Asmussen and Albrecher [1] presented excellent reviews about this well-known and important model.

In model (1), independent structures are usually assumed. For example, the claim amount $\left\{Y_{i},i\geq 1\right\}$ is a sequence of non-negative independent and identically distributed (i.i.d.) random variables, and the claim numbers of different periods are assumed to be a sequence of i.i.d. random variables. However, these are not always true in practice because of the increasing complexity of individual risks. To avoid this restriction, a growing number of actuaries have been paying attention to the model with dependent risks. As stated in Yang and Zhang [2], there are mainly two kinds of correlation in insurance: one is the correlation among lines of businesses, and the other is temporal dependence, such as the correlation between the current claim and the previous claims. For recent works about the first type of correlation, Refs. [3,4] studied the dependence among individual risks, Refs. [5,6] discussed the two-dimensional risk models with dependent surplus processes, and [7,8] examined the risk models that have multiple classes of insurance business with thinning dependence structure. The relevant results have been used in a variety of actuarial areas, including, among others, value at risk, dividend strategies, reinsurance, capital allocation, etc.

In this paper, we focus on the second type. To deal with this problem, the use of a time series is a critical method. Gerber [9] considered the calculation of ruin probabilities in a Gaussian linear risk model; Gourieroux and Jasiak [10] applied the integer-valued time series model to update the premiums in vehicle insurance; and many researchers have extensively revisited the relevant results afterwards. Considering that the compound distributions are the cornerstones of a great number of risk models in risk theory, Cossette et al. [11] proposed some new discrete-time risk models, where the first-order integer-valued moving average (INMA(1)) and first-order integer-valued autoregressive (INAR(1)) processes are used to describe the dependence structures among the number of claims for each period. The authors derived expressions for the functions that allow people to find the Lundberg adjustment coefficients and discussed the Lundberg approximation formulas for infinite-time ruin probabilities. Along the same line, Cossette et al. [12] determined the distributions of aggregate claim amount and provided an effective way to measure some related risk quantities, including VaR, TVaR, and the stop-loss premium. Shi and Wang [13] gave an approximation method for the risk model with the Poisson INAR(1) claim number process in order to obtain the upper bound of the infinite-time ruin probability. Zhang et al. [14] solved the problem of optimal reinsurance strategy for the risk model with the INMA(1) claim number process. Afterwards, Hu et al. [15] and Chen and Hu [16] further generalized this kind of model by replacing the Poisson innovations with compound Poisson innovations in the INAR(1) and INMA(1) claim number processes, respectively. Guan and Hu [17] even utilized an INAR(1) process with an arbitrary innovations’ distribution to specify the temporal dependence among the claim numbers.

In the papers mentioned above, it should be noted that the incomes of all the risk models are linear functions of time *t*, because the premiums are collected continuously with positive deterministic constant rate *c*, providing great convenience for risk analysis. However, this assumption is obviously lacking in terms of describing the real situation of insurance portfolios; for example, it cannot capture the uncertainty of the customers’ arrivals. As an alternative to a fixed premium rate, Boikov [18] supposesed that the premium income also follows a compound Poisson process and calculates the ruin probability. From then on, the risk models with stochastic premiums have been extensively improved by many actuaries. Wang et al. [19] studied the investment problem of such models. Labbé and Sendova [20] discussed the Gerber–Shiu function. Zhao and Yin [21] proposed a renewal risk model with stochastic incomes. Recently, Su et al. [22] provided a statistical method for estimating the Gerber–Shiu function; Ragulina [23] investigated the De Vylder approximation for the ruin probability and a constant dividend strategy in the risk model with stochastic premiums; and Dibu and Jacob [24] focused on a double barrier hybrid dividend strategy. Wang et al. [25] quantitatively assessed the impact of the stochastic income process on some ruin quantities in detail.

Similar to the classical risk model, the premium numbers of different periods are commonly set to be a sequence of i.i.d. random variables in the aforementioned papers. To better characterize the uncertainty and capture the variability of an insurer’s income process, Guan and Wang [26] proposed modeling the temporal dependence among the premium numbers of each period by a Poisson INAR(1) process. In this paper, we follow this trend of research. We also aim to study a new dependent risk model with stochastic premiums based on time series for count random variables, in which the INAR(1) process and INMA(1) process are applied to fit the temporal dependence among the premium numbers and the temporal dependence among the claim numbers of consecutive periods, respectively. Our goal is to approximate the infinite-time ruin probability of the proposed surplus process by the Lundberg adjustment coefficient and discuss the asymptotic formula for the finite-time ruin probability when the claim sizes follow distributions with heavy tails.

Our model generalizes the classical discrete-time surplus process of an insurance portfolio with stochastic premiums to a new dependent risk model, and our results extend what has been studied in the existing literature. The contributions of our paper mainly include the following two aspects:

- In contrast to the assumption that either claim numbers or premium numbers have a temporally dependent structure, we propose a new risk model of an insurance portfolio with both claim numbers and premium numbers being dependent within the integer-valued time series framework, which is more flexible in insurance practice.
- In addition to studying the distribution of the aggregate claims, the Lundberg adjustment coefficient, and the Lundberg approximation formula for the infinite-time ruin probability in the case of light-tailed claim sizes, we also explore the large deviations of the aggregate claims and the asymptotic formula for the finite-time ruin probability when the claim sizes are heavy-tailed, which enlarges the applicability of the risk model.

The remainder of the paper is organized as follows: Section 2 introduces our concerned risk model and considers some probabilistic properties of the proposed model. Section 3 defines the Lundberg adjustment coefficient via the solution of an explicit equation. Section 4 establishes an exponential asymptotic estimation for the infinite-time ruin probability. Section 5 studies the large deviations of the aggregate claims when the claim sizes follow a class of heavy-tailed distributions and presents an asymptotic formula for the finite-time ruin probability. Section 6 illustrates the main results by numerical simulations. Section 7 finally concludes this paper.

## 2. Risk Model and Basic Properties

In this section, we first describe the new dependent risk model, and then, provide some moment results of the premiums and claims. Let $U_{t}$ be the surplus of an insurance portfolio at the end of period *t*, and we define the surplus process by the dynamic equation

(2) $$\begin{array}{c} U_{t}=U_{t-1}+P_{t}-L_{t}=U_{t-1}+\sum_{k=1}^{M_{t}}X_{t,k}-\sum_{j=1}^{N_{t}}Y_{t,j},\;\;t=1,2,\cdots , \end{array}$$

where $U_{0}=u\geq 0$ is the initial surplus level; $P_{t}=\sum_{k=1}^{M_{t}}X_{t,k}$ aggregates the premiums during period *t*, in which $M_{t}$ counts the number of individual income and $X_{t,k}$ represents the amount of the *k*th premium income for the insurance portfolio during period *t*; $L_{t}=\sum_{j=1}^{N_{t}}Y_{t,j}$ is the aggregate claims during period *t*, in which $N_{t}$ denotes the number of claims and $Y_{t,j}$ is the size of the *j*th payment to the insured in period *t*. For mathematical tractability, the following assumptions are made:

(1) Both $\left\{X_{t,k},t=1,2,\cdots ,k=1,2,\cdots \right\}$ and $\left\{Y_{t,j},j=1,2,\cdots ,k=1,2,\cdots \right\}$ are arrays of i.i.d. random variables, which have the same distributions as non-negative *X* and *Y*, respectively.

(2) $\left\{X_{t,k},t=1,2,\cdots ,k=1,2,\cdots \right\}$, $\left\{Y_{t,j},j=1,2,\cdots ,k=1,2,\cdots \right\}$, $\left\{M_{t},t=1,2,\cdots \right\}$, and $\left\{N_{t},t=1,2,\cdots \right\}$ are mutually independent.

The dependence structures of the model are constructed in the following ways:

(i) $\left\{M_{t},t=1,2,\cdots \right\}$ constitutes a Poisson INAR(1) process that satisfies

(3) $$\begin{array}{c} M_{t}=\alpha \circ M_{t-1}+\varepsilon_{t},\;\;t=2,3,\cdots , \end{array}$$

where the so-called binomial thinning operator “∘” is given by

(4) $$\begin{array}{c} \alpha \circ M_{t-1}=\sum_{m=1}^{M_{t-1}}B_{t,m}^{\left(1\right)},\;\;t=2,3,\cdots , \end{array}$$

in which the following statements are true:

- The thinning parameter α∈[0,1).
- $\left\{B_{t,m}^{\left(1\right)},t=2,3,\cdots ,m=1,2,\cdots \right\}$ is an array of i.i.d. Bernoulli random variables with mean α.
- $\left\{\varepsilon_{t},t=2,3,\cdots \right\}$ is a sequence of i.i.d. Poisson random variables with mean $\lambda_{1}$.
- $M_{1}$, $\left\{B_{t,m}^{\left(1\right)},t=2,3,\cdots ,m=1,2,\cdots \right\}$ and $\left\{\varepsilon_{t},t=2,3,\cdots \right\}$ are independent.

(ii) $\left\{N_{t},t=1,2,\cdots \right\}$ constitutes a Poisson INMA(1) process that satisfies

(5) $$\begin{array}{c} N_{t}=\beta \circ \eta_{t-1}+\eta_{t},\;\;t=1,2,\cdots , \end{array}$$

where “∘” is similarly defined by

(6) $$\begin{array}{c} \beta \circ \eta_{t-1}=\sum_{m=1}^{\eta_{t-1}}B_{t,m}^{\left(2\right)},\;\;t=1,2,\cdots , \end{array}$$

in which the following are true:

- The thinning parameter β∈[0,1).
- $\left\{B_{t,m}^{\left(2\right)},t=1,2,\cdots ,m=1,2,\cdots \right\}$ is an array of i.i.d. Bernoulli random variables with mean β.
- $\left\{\eta_{t},t=0,1,\cdots \right\}$ is a sequence of i.i.d. Poisson random variables with mean $\lambda_{2}$.
- $\left\{B_{t,m}^{\left(2\right)},t=1,2,\cdots ,m=1,2,\cdots \right\}$ and $\left\{\eta_{t},t=1,2,\cdots \right\}$ are independent.

**Remark** **1.** *Time series analysis is one of the most important methods for dealing with dependent data and has attracted a lot of interest during the last decades. However, the classical real-valued time series models with continuous ranges can not account for discreteness, so they are of limited use for fitting the premium numbers and the claim numbers, which are typical count random variables fairly common in practice. Their poor performances in modeling this class of data mainly include: (1) the data generating mechanism can not be explained; (2) the approximate errors are big; and (3) the forecast results are not integer-valued. Therefore, models and methods for integer-valued time series have been covered by a large number of papers in recent years. Refs. [27,28,29,30] present comprehensive surveys on this fascinating research area. As two core models of integer-valued time series, INAR(1) process and INMA(1) process have been extensively applied in the literature of actuarial science, and the relevant results have been widely used in a variety of risk management.*

**Remark** **2.** *The INAR(1) process (3) shows that the premium number in period t is composed of two parts: $\varepsilon_{t}$ denotes the new incomes arriving between period t−1 and t, and $\alpha \circ M_{t-1}$ presents a random proportion of the premium number in the previous period. This can be reasonably explained for the insurance practice that states: every insured entity could continue to pay a premium with probability α; or withdraw from the contract with probability 1−α in the next period. When α=0, (3) becomes $M_{t}=\varepsilon_{t}$, meaning that the premium number in period t could be totally determined by $\varepsilon_{t}$, and our model (2) will reduce to the classical discrete-time risk model with stochastic premiums, where the premium numbers of different periods are independent (please see Appendix A for details).*

**Remark** **3.** *The INMA(1) process (5) reveals that the claim number in period t also consists of two parts: $\eta_{t}$ is the new claim during period t, and $\beta \circ \eta_{t-1}$ indicates the claims of period t−1 that could produce another accident with probability β in period t. Instead of (3), we use the INMA(1) process (5) to fit the temporal dependence among the claim numbers for each period, considering that the insured parties cannot receive benefits every year for some insurance products. Taking unemployment insurance as an example, every time the claimant is out of work, they could receive the benefits for up to 2 years, if the premiums for at least 1 year have been paid. Another appropriate example might be some medical insurance contracts, which state that no matter how long the patient stays in the hospital, the insurer would pay the benefits for at most (for instance) 2 months. Similarly, if β=0, our proposed model will reduce to the classical case, where the claim numbers of different periods are independent.*

As stated in Al-Osh and Alzaid [31], under the condition of 0≤α<1, it follows that the process of premium numbers $\left\{M_{t},t=1,2,\cdots \right\}$ is a stationary and ergodic Markov chain. Furthermore, if we assume $\varepsilon_{t}∼P\left(\lambda_{1}\right)$, then $M_{t}$ is also Poisson distributed with mean $\frac{\lambda_{1}}{1-\alpha }$. Hence, by the law of iterated expectation and the assumption that $\left\{X_{t,k},k=1,2,\cdots \right\}$ and $M_{t}$ are independent, it is easy to find that

(7) $$\begin{array}{c} E(P_{t}=E\left[E\left(P_{t}|M_{t}\right)\right]=E\left[E\left(\sum_{k=1}^{M_{t}}X_{t,k}|M_{t}\right)\right]=E\left[\sum_{k=1}^{M_{t}}E\left(X_{t,k}|M_{t}\right)\right]=E\left[\sum_{k=1}^{M_{t}}E\left(X_{t,k}\right)\right]=E\left(M_{t}\right)E\left(X\right)=\frac{\lambda_{1}}{1-\alpha }E\left(X\right). \end{array}$$

Meanwhile, by the law of total variance, we can obtain

(8) $$\begin{array}{cc} \mathrm{Var}(P_{t})\; & =\mathrm{Var}\left[E\left(P_{t}|M_{t}\right)\right]+E\left[\mathrm{Var}\left(P_{t}|M_{t}\right)\right]\\ \; & =\mathrm{Var}\left[E\left(\sum_{k=1}^{M_{t}}X_{t,k}|M_{t}\right)\right]+E\left[\mathrm{Var}\left(\sum_{k=1}^{M_{t}}X_{t,k}|M_{t}\right)\right]\\ \; & =\mathrm{Var}\left[\sum_{k=1}^{M_{t}}E\left(X_{t,k}|M_{t}\right)\right]+E\left[\sum_{k=1}^{M_{t}}\mathrm{Var}\left(X_{t,k}|M_{t}\right)\right]\\ \; & =\mathrm{Var}\left[\sum_{k=1}^{M_{t}}E\left(X_{t,k}\right)\right]+E\left[\sum_{k=1}^{M_{t}}\mathrm{Var}\left(X_{t,k}\right)\right]\\ \; & =\mathrm{Var}\left[M_{t}\cdot E\left(X\right)\right]+E\left[M_{t}\cdot \mathrm{Var}\left(X\right)\right]\\ \; & =E\left(M_{t}\right)\mathrm{Var}\left(X\right)+\mathrm{Var}\left(M_{t}\right){\left[E\left(X\right)\right]}^{2}=\frac{\lambda_{1}}{1-\alpha }E\left(X^{2}\right). \end{array}$$

Furthermore, Al-Osh and Alzaid [31] show that

$$\begin{array}{c} \mathrm{Cov}\left(M_{t},M_{t+h}\right)=\alpha^{h}\mathrm{Var}\left(M_{t}\right)=\frac{\lambda_{1}\alpha^{h}}{1-\alpha }, \end{array}$$

from which we can obtain

(9) $$\begin{array}{cc} \mathrm{Cov}(P_{t},P_{t+h})\; & =E\left(P_{t}P_{t+h}\right)-E\left(P_{t}\right)E\left(P_{t+h}\right)\\ \; & =E\left[E\left(P_{t}P_{t+h}|M_{t},M_{t+h}\right)\right]-E\left(M_{t}\right)E\left(M_{t+h}\right){\left[E\left(X\right)\right]}^{2}\\ \; & =E\left[E\left(P_{t}|M_{t}\right)E\left(P_{t+h}|M_{t+h}\right)\right]-E\left(M_{t}\right)E\left(M_{t+h}\right){\left[E\left(X\right)\right]}^{2}\\ \; & =E\left(M_{t}M_{t+h}\right){\left[E\left(X\right)\right]}^{2}-E\left(M_{t}\right)E\left(M_{t+h}\right){\left[E\left(X\right)\right]}^{2}\\ \; & ={\left[E\left(X\right)\right]}^{2}\mathrm{Cov}\left(M_{t},M_{t+h}\right)=\frac{\lambda_{1}\alpha^{h}}{1-\alpha }{\left[E\left(X\right)\right]}^{2},\;\;h=1,2,\cdots . \end{array}$$

Similarly, for the process of claim numbers $\left\{N_{t},t=1,2,\cdots \right\}$, under the condition of 0≤β<1, its marginal distribution is uniquely determined by the law of $\left\{\eta_{t},t=0,1,\cdots \right\}$. Therefore, the assumption of $\eta_{t}∼P\left(\lambda_{2}\right)$ will result in $N_{t}$ being Poisson distributed with a mean of $\left(1+\beta \right)\lambda_{2}$. Consequently, by the same method to drive (7)–(9), we have

$$\begin{array}{c} E\left(L_{t}\right)=E\left(N_{t}\right)E\left(Y\right)=\left(1+\beta \right)\lambda_{2}E\left(Y\right), \end{array}$$

$$\begin{array}{c} \mathrm{Var}\left(L_{t}\right)=E\left(N_{t}\right)\mathrm{Var}\left(Y\right)+\mathrm{Var}\left(N_{t}\right){\left[E\left(Y\right)\right]}^{2}=\left(1+\beta \right)\lambda_{2}E\left(Y^{2}\right). \end{array}$$

and

(10) $$\begin{array}{c} \mathrm{Cov}\left(L_{t},L_{t+h}\right)={\left[E\left(Y\right)\right]}^{2}\mathrm{Cov}\left(N_{t},N_{t+h}\right)=\left\{\begin{array}{cc} \lambda_{2}\beta {\left[E\left(Y\right)\right]}^{2}, & h=1,\\ 0, & h>1. \end{array}\right. \end{array}$$

These results are consistent with those in [11].

## 3. Definition of the Lundberg Adjustment Coefficient

In this section, we first consider how to calculate the moment generating functions (m.g.f.) of the aggregate premiums and aggregate claims up to period *t*, and then, define the Lundberg adjustment coefficient of the proposed dependent risk model with stochastic premiums based on time series for count random variables by means of a equation.

After recursive calculation, we can rewrite model (2) as

(11) $$\begin{array}{cc} U_{t}=U_{t-1}+P_{t}-L_{t}\; & =U_{t-1}+\sum_{k=1}^{M_{t}}X_{t,k}-\sum_{j=1}^{N_{t}}Y_{t,j}\\ \; & =u+\sum_{i=1}^{t}\sum_{k=1}^{M_{i}}X_{i,k}-\sum_{i=1}^{t}\sum_{j=1}^{N_{i}}Y_{i,j}=u+W_{t}-S_{t},\;\;t=1,2,\cdots , \end{array}$$

in which $W_{t}=\sum_{i=1}^{t}P_{i}=\sum_{i=1}^{t}\sum_{k=1}^{M_{i}}X_{i,k}$ and $S_{t}=\sum_{i=1}^{t}L_{i}=\sum_{i=1}^{t}\sum_{j=1}^{N_{i}}Y_{i,j}$ represent the aggregate premium incomes and aggregate claim payments up to time *t*, respectively. As for the m.g.f. of $W_{t}$ and $S_{t}$, by the definition, we have that

(12) $$\begin{array}{cc} M_{W_{t}}\left(r\right)\; & =E(e^{rW_{t}})\\ \; & =E[e^{r(P_{1}+\cdots +P_{t})}]\\ \; & =M_{P_{1},\cdots ,P_{t}}\left(r,\cdots ,r\right)\\ \; & =P_{M_{1},\cdots ,M_{t}}\left(M_{X}\left(r\right),\cdots ,M_{X}\left(r\right)\right)\\ \; & =E[M_{X}{\left(r\right)}^{M_{1}}\cdots M_{X}{\left(r\right)}^{M_{t}}]\\ \; & =P_{M_{1}+\cdots +M_{t}}\left(M_{X}\left(r\right)\right), \end{array}$$

where $M_{X}\left(\cdot \right)$ denotes the m.g.f. of *X* and $P_{M_{1}+\cdots +M_{t}}\left(\cdot \right)$ presents the probability generating function (p.g.f.) of the total premium number up to period *t* of the proposed model (2).

Similarly, it holds that

(13) $$\begin{array}{c} M_{S_{t}}\left(r\right)=P_{N_{1}+\cdots +N_{t}}\left(M_{Y}\left(r\right)\right), \end{array}$$

where $M_{Y}\left(\cdot \right)$ denotes the m.g.f. of *Y*, and $P_{N_{1}+\cdots +N_{t}}\left(\cdot \right)$ presents the p.m.f. of the total claim number up to period *t* of the proposed model (2).

In order to compute $M_{W_{t}}\left(r\right)$ and $M_{S_{t}}\left(r\right)$, we find the explicit expressions for $P_{M_{1}+\cdots +M_{t}}\left(\cdot \right)$ and $P_{N_{1}+\cdots +N_{t}}\left(\cdot \right)$ in the following two lemmas, respectively.

**Lemma** **1.** *For t=1,2,⋯, when 0≤s≤1, the p.g.f. of $M_{1}+\cdots +M_{t}$ is given by*

(14) $$\begin{array}{c} P_{M_{1}+\cdots +M_{t}}\left(s\right)=\exp\left\{\lambda \frac{s-1}{1-\alpha s}\left[t+\frac{1-{\left(\alpha s\right)}^{t}}{1-\alpha }-\frac{1-{\left(\alpha s\right)}^{t}}{1-\alpha s}\right]\right\}. \end{array}$$

**Proof.**  Since $M_{1}∼P\left(\frac{\lambda_{1}}{1-\alpha }\right)$, it is obvious that

$$\begin{array}{c} P_{M_{1}}\left(s\right)=\exp\left\{\frac{\lambda_{1}}{1-\alpha }\left(s-1\right)\right\}. \end{array}$$

When t≥2, we denote

$$\begin{array}{c} \alpha^{\left(t\right)}\circ M_{1}=\underset{t-\mathrm{fold}\;\mathrm{operation}}{\underset{︸}{\alpha \circ \cdots \circ \alpha \circ }}M_{1}. \end{array}$$

By the property of the binomial thinning operator (see Scotto et al. [28] for example), we can rewrite $M_{1}+\cdots +M_{t}$ as

(15) $$\begin{array}{cc} M_{1}+\cdots +M_{t}\; & =M_{1}+\alpha \circ M_{1}+\alpha^{\left(2\right)}\circ M_{1}+\cdots +\alpha^{\left(t-1\right)}\circ M_{1}\\ \; & \;\;+\varepsilon_{2}+\alpha \circ \varepsilon_{2}+\cdots +\alpha^{\left(t-2\right)}\circ \varepsilon_{2}\\ \; & \;\;+\cdots \\ \; & \;\;+\varepsilon_{t-1}+\alpha \circ \varepsilon_{t-1}\\ \; & \;\;+\varepsilon_{t}. \end{array}$$

For the p.g.f. calculation, we have

(16) $$\begin{array}{cc} P_{M_{1}+\alpha \circ M_{1}+\alpha^{\left(2\right)}\circ M_{1}+\cdots +\alpha^{\left(t-1\right)}\circ M_{1}}\left(s\right)\; & =E(s^{M_{1}+\alpha \circ M_{1}+\alpha^{\left(2\right)}\circ M_{1}+\cdots +\alpha^{\left(t-1\right)}\circ M_{1}})\\ \; & =E\left[s^{M_{1}}s^{\alpha \circ M_{1}}\cdots s^{\alpha^{\left(t-2\right)}\circ M_{1}}E\left(s^{\alpha^{\left(t-1\right)}\circ M_{1}}|M_{1},\cdots ,\alpha^{\left(t-2\right)}\circ M_{1}\right)\right]\\ \; & =E\left[s^{M_{1}}s^{\alpha \circ M_{1}}\cdots s^{\alpha^{\left(t-2\right)}\circ M_{1}}{\left(\alpha s+1-\alpha \right)}^{\alpha^{\left(t-2\right)}\circ M_{1}}\right]\\ \; & =E\left[s^{M_{1}}s^{\alpha \circ M_{1}}\cdots s^{\alpha^{\left(t-3\right)}\circ M_{1}}{\left(h_{2}\left(s\right)\right)}^{\alpha^{\left(t-2\right)}\circ M_{1}}\right]\\ \; & =E\left[s^{M_{1}}s^{\alpha \circ M_{1}}\cdots s^{\alpha^{\left(t-3\right)}\circ M_{1}}{\left(\alpha h_{2}\left(s\right)+1-\alpha \right)}^{\alpha^{\left(t-3\right)}\circ M_{1}}\right]\\ \; & =E\left[s^{M_{1}}s^{\alpha \circ M_{1}}\cdots s^{\alpha^{\left(t-4\right)}\circ M_{1}}{\left(h_{3}\left(s\right)\right)}^{\alpha^{\left(t-3\right)}\circ M_{1}}\right]\\ \; & =\cdots \\ \; & =E\left[s^{M_{1}}{\left(\alpha h_{t-1}\left(s\right)+1-\alpha \right)}^{\alpha \circ M_{1}}\right]\\ \; & =\exp\left\{\frac{\lambda_{1}}{1-\alpha }\left(h_{t}\left(s\right)-1\right)\right\}, \end{array}$$

in which $h_{1}\left(s\right)=s$ and $h_{t}\left(s\right)=s\left(\alpha h_{t-1}\left(s\right)+1-\alpha \right)$.

Similarly, we can obtain

(17) $$\begin{array}{c} P_{\varepsilon_{2}+\alpha \circ \varepsilon_{2}+\cdots +\alpha^{\left(t-2\right)}\circ \varepsilon_{2}}\left(s\right)=\exp\left\{\lambda_{1}\left(h_{t-1}\left(s\right)-1\right)\right\},\cdots ,P_{\varepsilon_{t}}\left(s\right)=\exp\left\{\lambda_{1}\left(h_{1}\left(s\right)-1\right)\right\}. \end{array}$$

Combining (15)–(17), it follows that

(18) $$\begin{array}{cc} \; & P_{M_{1}+\cdots +M_{t}}\left(s\right)\\ = & P_{M_{1}+\alpha \circ M_{1}+\alpha^{\left(2\right)}\circ M_{1}+\cdots +\alpha^{\left(t-1\right)}\circ M_{1}}\left(s\right)\times P_{\varepsilon_{2}+\alpha \circ \varepsilon_{2}+\cdots +\alpha^{\left(t-2\right)}\circ \varepsilon_{2}}\left(s\right)\times \cdots \times P_{\varepsilon_{t-1}+\alpha \circ \varepsilon_{t-1}}\left(s\right)\times P_{\varepsilon_{t}}\left(s\right)\\ = & \exp\left\{\frac{\lambda_{1}}{1-\alpha }\left(h_{t}\left(s\right)-1\right)\right\}\prod_{i=1}^{t-1}\exp\left\{\lambda_{1}\left(h_{i}\left(s\right)-1\right)\right\}. \end{array}$$

Moreover, from the definition $h_{t}\left(s\right)=s\left(\alpha h_{t-1}\left(s\right)+1-\alpha \right)$, it is easy to find that

$$h_{t}\left(s\right)-1=s-1+\alpha s\left(h_{t-1}\left(s\right)-1\right).$$

Then, recursive calculation results in

(19) $$h_{t}\left(s\right)-1=\left(s-1\right)\frac{1-{\left(\alpha s\right)}^{t}}{1-\alpha s}.$$

Finally, inserting (19) into (18), we can obtain

$$\begin{array}{c} P_{M_{1}+\cdots +M_{t}}\left(s\right)=\exp\left\{\lambda \frac{s-1}{1-\alpha s}\left[t+\frac{1-{\left(\alpha s\right)}^{t}}{1-\alpha }-\frac{1-{\left(\alpha s\right)}^{t}}{1-\alpha s}\right]\right\}. \end{array}$$

This completes the proof. □

**Lemma** **2.** *For t=1,2,⋯, when s≥0, the p.g.f. of $N_{1}+\cdots +N_{t}$ is given by*

(20) $$\begin{array}{c} P_{N_{1}+\cdots +N_{t}}\left(s\right)=\exp\left\{\lambda_{2}\left(1+\beta \right)\left(s-1\right)+\lambda_{2}\left(t-1\right)\left[\beta s^{2}+\left(1-\beta \right)s-1\right]\right\}. \end{array}$$

**Proof.**  By (5), it holds that

$$\begin{array}{cc} P_{N_{1}+\cdots +N_{t}}\left(s\right)\; & =E(s^{N_{1}+\cdots +N_{t}})\\ \; & =E(s^{\beta \circ \eta_{0}+\eta_{1}}s^{\beta \circ \eta_{1}+\eta_{2}}\cdots s^{\beta \circ \eta_{t-1}+\eta_{t}})\\ \; & =E(s^{\beta \circ \eta_{0}}s^{\eta_{1}+\beta \circ \eta_{1}}\cdots s^{\eta_{t-1}+\beta \circ \eta_{t-1}}s^{\eta_{t}})\\ \; & =E\left(s^{\beta \circ \eta_{0}}\right)E\left(s^{\eta_{1}+\beta \circ \eta_{1}}\right)\cdots E\left(s^{\eta_{t-1}+\beta \circ \eta_{t-1}}\right)E\left(s^{\eta_{t}}\right)\\ \; & =\exp\left\{\lambda_{2}\left(1+\beta \right)\left(s-1\right)+\lambda_{2}\left(t-1\right)\left[\beta s^{2}+\left(1-\beta \right)s-1\right]\right\}, \end{array}$$

which follows from $\eta_{t}∼P\left(\lambda_{2}\right)$, $\beta \circ \eta_{0}∼P\left(\beta \lambda_{2}\right)$ and

$$\begin{array}{cc} P_{\varepsilon_{i}+\beta \circ \varepsilon_{i}}\left(s\right)\; & =E(s^{\varepsilon_{i}+\beta \circ \varepsilon_{i}})\\ \; & =E[E\left(s^{\varepsilon_{i}}s^{\beta \circ \varepsilon_{i}}|\varepsilon_{i}\right)]\\ \; & =E[s^{\varepsilon_{i}}E\left(s^{\beta \circ \varepsilon_{i}}|\varepsilon_{i}\right)]\\ \; & =E[s^{\varepsilon_{i}}{\left(\beta s+1-\beta \right)}^{\varepsilon_{i}}]\\ \; & =\exp\left\{\lambda_{2}\left[\beta s^{2}+\left(1-\beta \right)s-1\right]\right\},\;\;i=1,2,\cdots ,t-1. \end{array}$$

The proof then is completed. □

To further analyze the insurance portfolio, we write

(21) $$\begin{array}{c} c_{t}\left(r\right)=\frac{1}{t}\ln E\left(\left[e^{r(S_{t}-W_{t})}\right]\right), \end{array}$$

and let

(22) $$\begin{array}{c} c\left(r\right)=\lim_{t\to +\infty }c_{t}\left(r\right). \end{array}$$

Then, the positive solution to the equation c(r)=0 can be defined as the Lundberg adjustment coefficient, which is denoted by *R* and can be used to approximate the infinite-time ruin probability of the proposed model (2). The following result gives the explicit expression for c(r).

**Theorem** **1.** *For r≥0, we have*

(23) $$\begin{array}{c} c\left(r\right)=\lambda_{1}\frac{M_{X}\left(-r\right)-1}{1-\alpha M_{X}\left(-r\right)}+\lambda_{2}\left[\beta M_{Y}^{2}\left(r\right)+\left(1-\beta \right)M_{Y}\left(r\right)-1\right]. \end{array}$$

**Proof.**  Due to the non-negativity of *r* and *X*, it follows that

$$0\leq M_{X}\left(-r\right)\leq 1,\;0\leq \alpha M_{X}\left(-r\right)<1.$$

Then, by Lemma 1, we have

(24) $$\begin{array}{cc} \lim_{t\to +\infty }\frac{1}{t}\ln E\left(e^{-rW_{t}}\right)\; & =\lim_{t\to +\infty }\frac{1}{t}\ln P_{M_{1}+\cdots +M_{t}}\left(M_{X}\left(-r\right)\right)\\ \; & =\lim_{t\to +\infty }\frac{1}{t}\ln\left(\exp\left\{\lambda \frac{M_{X}\left(-r\right)-1}{1-\alpha M_{X}\left(-r\right)}\left[t+\frac{1-{\left(\alpha M_{X}\left(-r\right)\right)}^{t}}{1-\alpha }-\frac{1-{\left(\alpha M_{X}\left(-r\right)\right)}^{t}}{1-\alpha M_{X}\left(-r\right)}\right]\right\}\right)\\ \; & =\lambda \frac{M_{X}\left(-r\right)-1}{1-\alpha M_{X}\left(-r\right)}. \end{array}$$

On the other hand, from (13) and (20), we obtain

(25) $$\begin{array}{cc} \lim_{t\to +\infty }\frac{1}{t}\ln E\left(e^{rS_{t}}\right)\; & =\lim_{t\to +\infty }\frac{1}{t}\ln P_{N_{1}+\cdots +N_{t}}\left(M_{Y}\left(r\right)\right)\\ \; & =\lim_{t\to +\infty }\frac{1}{t}\ln\left(\exp\left\{\lambda_{2}\left(1+\beta \right)\left(M_{Y}\left(r\right)-1\right)+\lambda_{2}\left(t-1\right)\left[\beta M_{Y}^{2}\left(r\right)+\left(1-\beta \right)M_{Y}\left(r\right)-1\right]\right\}\right)\\ \; & =\lambda_{2}\left(1+\beta \right)\lim_{t\to +\infty }\frac{1}{t}\left(M_{Y}\left(r\right)-1\right)+\lim_{t\to +\infty }\frac{t-1}{t}\lambda_{2}\left[\beta M_{Y}^{2}\left(r\right)+\left(1-\beta \right)M_{Y}\left(r\right)-1\right]\\ \; & =\lambda_{2}\left[\beta M_{Y}^{2}\left(r\right)+\left(1-\beta \right)M_{Y}\left(r\right)-1\right]. \end{array}$$

Then, combining (24) and (25) with (21) and (22) yields

$$\begin{array}{cc} c(r)\; & =\lim_{t\to +\infty }c_{t}\left(r\right)\\ \; & =\lim_{t\to +\infty }\frac{1}{t}\ln E\left(\left[e^{r(S_{t}-W_{t})}\right]\right)\\ \; & =\lim_{t\to +\infty }\frac{1}{t}\ln E\left(e^{rS_{t}}\right)+\lim_{t\to +\infty }\frac{1}{t}\ln E\left(e^{-rW_{t}}\right)\\ \; & =\lambda_{1}\frac{M_{X}\left(-r\right)-1}{1-\alpha M_{X}\left(-r\right)}+\lambda_{2}\left[\beta M_{Y}^{2}\left(r\right)+\left(1-\beta \right)M_{Y}\left(r\right)-1\right], \end{array}$$

This completes the proof. □

**Remark** **4.** *When α=0, the proposed model (2) degenerates to the discrete-time risk model based on the Poisson INMA(1) process studied by [11,14], where only the temporal dependence among the claim numbers of consecutive periods is considered. Consequently, (23) becomes*

$$c\left(r\right)=\lambda_{1}\left[M_{X}\left(-r\right)-1\right]+\lambda_{2}\left[\beta M_{Y}^{2}\left(r\right)+\left(1-\beta \right)M_{Y}\left(r\right)-1\right],$$

*which corresponds to (7) in [11,14].*

**Remark** **5.** *When β=0, the proposed model (2) reduces to the discrete-time risk model with stochastic premiums and dependence based on the Poisson INAR(1) process studied by [26], where only the temporal dependence among the premium numbers of consecutive periods is considered. As a result, (23) becomes*

$$c\left(r\right)=\lambda_{1}\frac{M_{X}\left(-r\right)-1}{1-\alpha M_{X}\left(-r\right)}+\lambda_{2}\left[M_{Y}\left(r\right)-1\right],$$

*which corresponds to (3.10) in [26].*

## 4. Lundberg Approximation Formula for the Infinite-Time Ruin Probability

Let the ruin time of our proposed surplus process (2) be $T=\underset{t\in \{0,1,2,\cdots \}}{\inf}\left\{t,U_{t}\leq 0\right\}$ if $U_{t}$ goes below 0 at least once; otherwise, take T=+∞. As a consequence, the infinite-time ruin probability ψ(u) is defined by

$$\begin{array}{c} \psi \left(u\right)=P\left(T<+\infty |U_{0}=u\right). \end{array}$$

Ruin probability ψ(u) is well-known as one of the most common and important quantities used to measure the riskiness of an insurance portfolio in the risk-theoretic context. However, as can be seen from the expression (11), our proposed model releases the condition that $P_{t}$ and $L_{t}$ are independent of $U_{t-1}$, which is a key but defective assumption in the classical risk model with stochastic premiums and allows for the temporal dependence among the premium numbers and claim numbers. Therefore, $P_{t}$ and $L_{t}$ are correlated with $U_{t-1}$, and $\left\{U_{t},t=1,2,\cdots \right\}$ is no longer a Lévy process with stationary independent increments in our model. Consequently, it is not easy to derive the upper bounds and explicit expression for the infinite-time ruin probability such as those in some classical models. As an efficient alternative, the following result gives an asymptotic estimation for ψ(u) of our proposed model (2).

**Theorem** **2.** *In the discrete-time dependent risk model with stochastic premiums based on the Poisson INAR(1) process and Poisson INMA(1) process, if*

(26) $$\begin{array}{c} \frac{\lambda_{1}}{1-\alpha }E\left(X\right)>\lambda_{2}\left(1+\beta \right)E\left(Y\right), \end{array}$$

*we can obtain the Lundberg approximation formula for the infinite-time ruin probability ψ(u), which has the following expression:*

(27) $$\begin{array}{c} \lim_{u\to +\infty }-\frac{\ln(\psi (u))}{u}=R, \end{array}$$

*where u and R are the initial capital and the Lundberg adjustment coefficient, respectively.*

**Proof.**  According to Theorem 2.1 in Müller and Pflug [32], it is sufficient for us to prove that the equation c(r)=0 has a unique positive solution, which can be defined as the Lundberg adjustment coefficient *R*. To this end, we derive the following four properties of the function c(r).

Firstly, noting that $M_{X}\left(0\right)=M_{Y}\left(0\right)=1$, we have

(28) $$\begin{array}{c} c\left(0\right)=\lambda_{1}\frac{M_{X}\left(0\right)-1}{1-\alpha M_{X}\left(0\right)}+\lambda_{2}\left[\beta M_{Y}^{2}\left(0\right)+\left(1-\beta \right)M_{Y}\left(0\right)-1\right]=0. \end{array}$$

Secondly, it is easy to calculate that

$$\begin{array}{c} c^{\prime }\left(r\right)=\frac{-\lambda_{1}\left(1-\alpha \right)M_{X}^{\prime }\left(-r\right)}{{\left[1-\alpha M_{X}\left(-r\right)\right]}^{2}}+\lambda_{2}\left[2\beta M_{Y}\left(r\right)M_{Y}^{\prime }\left(r\right)+\left(1-\beta \right)M_{Y}^{\prime }\left(r\right)\right]. \end{array}$$

Together with the fact $M_{X}^{\prime }\left(0\right)=E\left(X\right)$ and $M_{Y}^{\prime }\left(0\right)=E\left(Y\right)$, we obtain

(29) $$\begin{array}{cc} c^{\prime }\left(0\right)\; & =\frac{-\lambda_{1}\left(1-\alpha \right)M_{X}^{\prime }\left(0\right)}{{\left[1-\alpha M_{X}\left(0\right)\right]}^{2}}+\lambda_{2}\left[2\beta M_{Y}\left(0\right)M_{Y}^{\prime }\left(0\right)+\left(1-\beta \right)M_{Y}^{\prime }\left(0\right)\right]\\ \; & =\lambda_{2}\left(1+\beta \right)E\left(Y\right)-\frac{\lambda_{1}}{1-\alpha }E\left(X\right)<0. \end{array}$$

Thirdly, it is easy to verify the convexity of c(r), which results from the fact that $c_{t}\left(r\right)$ is convex and the definition of $c\left(r\right)=\lim_{t\to +\infty }c_{t}\left(r\right)$.

Finally, when the m.g.f. of *Y* exists, i.e., there exists some quantity $r_{0}$, $0<r_{0}\leq +\infty$, such that $M_{Y}\left(r\right)$ is finite for all $r<r_{0}$ with

$$\begin{array}{c} \lim_{r\to r_{0}^{-}}M_{Y}\left(r\right)=+\infty , \end{array}$$

then, it holds that

(30) $$\begin{array}{c} \lim_{r\to r_{0}^{-}}c\left(r\right)=\lim_{r\to r_{0}^{-}}\left(\lambda_{1}\frac{M_{X}\left(-r\right)-1}{1-\alpha M_{X}\left(-r\right)}+\lambda_{2}\left[\beta M_{Y}^{2}\left(r\right)+\left(1-\beta \right)M_{Y}\left(r\right)-1\right]\right)=+\infty . \end{array}$$

Therefore, it can be concluded that there exists a unique positive solution to the equation c(r)=0, and then, (27) follows immediately. □

**Remark** **6.** *In risk and ruin theory, the assumption (26) is the so-called relative safety loading condition, which implies that the expected premium incomes should be more than the expected claim expenses to guarantee that the insurance company can operate normally and profitably.*

**Remark** **7.** *As a result of the approximation formula (27), we can asymptotically estimate the infinite-time ruin probability ψ(u) by*

(31) $$\begin{array}{c} \psi \left(u\right)⋍e^{-Ru}, \end{array}$$

*if the initial surplus u becomes large enough.*

From (9) and (10), it can be seen that the thinning parameters α and β could quantitatively measure the degree of the dependence in the risk model (2); hence, it is necessary for us to discuss their impacts on the adjustment coefficient and further on the risk of the insurance portfolio.

**Proposition** **1.** *As a function of the two thinning parameters, the Lundberg adjustment coefficient R of our proposed risk model (2) increases with respect to α and decreases with respect to β.*

**Proof.**  For convenience, we now rewrite c(r) as c(α,β,r); the Lundberg adjustment coefficient *R* is determined by c(α,β,R)=0 and can be taken as a function of α and β. By the properties derived in the proof of Theorem 2, we know that

$$\begin{array}{c} \frac{\partial c(\alpha ,\beta ,R)}{\partial R}>0. \end{array}$$

Meanwhile, with R>0 in mind, it follows that $0\leq M_{X}\left(-R\right)<1$. Thus, we take the partial derivative of c(α,β,R) with respect to variable α and then have

$$\begin{array}{cc} \frac{\partial c(\alpha ,\beta ,R)}{\partial \alpha }\; & =\frac{\partial }{\partial \alpha }\left(\lambda_{1}\frac{M_{X}\left(-R\right)-1}{1-\alpha M_{X}\left(-R\right)}+\lambda_{2}\left[\beta M_{Y}^{2}\left(R\right)+\left(1-\beta \right)M_{Y}\left(R\right)-1\right]\right)\\ \; & =\frac{-\lambda_{1}M_{X}\left(-R\right)\left[1-\alpha M_{X}\left(-R\right)\right]+\lambda_{1}\left(1-\alpha \right)M_{X}\left(-R\right)M_{X}\left(-R\right)}{{\left[1-\alpha M_{X}\left(-R\right)\right]}^{2}}\\ \; & =\frac{\lambda_{1}\left[M_{X}^{2}\left(-R\right)-M_{X}\left(-R\right)\right]}{{\left[1-\alpha M_{X}\left(-R\right)\right]}^{2}}<0. \end{array}$$

As a result, using implicit function theorem, it holds that

$$\begin{array}{c} \frac{\partial R}{\partial \alpha }=-\frac{\left(\partial /\partial \alpha )c(\alpha ,\beta ,R\right)}{\left(\partial /\partial R)c(\alpha ,\beta ,R\right)}>0, \end{array}$$

implying that *R* increases with respect to α.

Similarly, because $M_{Y}\left(R\right)>1$ for R>0, taking the partial derivative of c(α,β,R) with respect to variable β yields

$$\begin{array}{cc} \frac{\partial c(\alpha ,\beta ,R)}{\partial \beta }\; & =\frac{\partial }{\partial \beta }\left(\lambda_{1}\frac{M_{X}\left(-R\right)-1}{1-\alpha M_{X}\left(-R\right)}+\lambda_{2}\left[\beta M_{Y}^{2}\left(R\right)+\left(1-\beta \right)M_{Y}\left(R\right)-1\right]\right)\\ \; & =\lambda_{2}\left[M_{Y}^{2}\left(R\right)-M_{Y}\left(R\right)\right]>0, \end{array}$$

from which we apply implicit function theorem again and obtain

$$\begin{array}{c} \frac{\partial R}{\partial \beta }=-\frac{\left(\partial /\partial \beta )c(\alpha ,\beta ,R\right)}{\left(\partial /\partial R)c(\alpha ,\beta ,R\right)}<0, \end{array}$$

meaning that *R* decreases with respect to β. □

**Remark** **8.** *As shown in Proposition 1, the degree of riskiness can be measured and quantified by the Lundberg adjustment coefficient R, in the sense that it decreases with the thinning parameter α, while it increases with the thinning parameter β. In insurance practice, it can be naturally explained that when α increases, the insured parties would like to renew their insurance contracts with a higher probability in the next period, which would lower the risk of the portfolio. On the contrary, when β increases, a reported claim becomes more likely to produce another insurance accident in the next period, which could make the portfolio much riskier.*

## 5. Asymptotic Formula for the Finite-Time Ruin Probability

In this section, we turn our focus to the case of heavy-tailed claim sizes, which are frequently used in insurance practice for catastrophe risks, such as earthquakes, hurricanes, floods, financial crises, agricultural disasters, and so on. In these instances, the Lundberg adjustment coefficient and Lundberg approximation estimation for infinite-time ruin probability can no longer be applied because $M_{Y}\left(r\right)$ (the m.g.f. of *Y*) does not exist for r>0. Therefore, increasing numbers of researchers have increasingly paid close attention to the precise large deviations in the aggregate of claims, as well as the asymptotic formulas for infinite-time and finite-time ruin probabilities. The relevant study was initiated by Klüppelberg and Mikosch [33] and then has been revisited by many researchers afterwards. We refer to Chen et al. [34] and Fu et al. [35] for some recent contributions on this topic. Cheng and Wang [36], Yang et al. [37], and Jing et al. [38] considered the asymptotic ruin probabilities in risk models with dependence among the claim sizes. Xun et al. [39] obtained the uniformly asymptotic result of ruin probability in a general risk model with stochastic premiums. Yu [40] derived the precise large deviations of the aggregate amount of claims for a risk model with the Poisson ARCH claim number process. Along the same line, in this section, we investigate our proposed model (2) when the distribution of claim sizes belongs to a heavy-tailed class.

First, we give some brief notations. Let a(x) and b(x) be two positive functions. We denote a(x)∼b(x) if $\lim_{x\to +\infty }a\left(x\right)/b\left(x\right)=1$; denote a(x)≲b(x) if $\underset{x\to +\infty }{lim sup}a\left(x\right)/b\left(x\right)\leq 1$; denote a(x)≳b(x) if $\underset{x\to +\infty }{lim inf}a\left(x\right)/b\left(x\right)\geq 1$; and denote a(x)=o(b(x)) if $\underset{x\to +\infty }{lim sup}a\left(x\right)/b\left(x\right)=0$. We denote the common distribution functions of premium amount *X* and claim size *Y* with $F_{X}\left(x\right)$ and $F_{Y}\left(y\right)$, respectively.

Then, we recall a class of heavy-tailed distributions and one of its important properties. More detailed discussions can be found in Embrechts et al. [41], Asmussen and Albrecher [1], etc.

A distribution function *F* on [0,∞] is said to have a consistently varying tail, denoted by F∈C, if

(32) $$\lim_{y↑1}\underset{x\to +\infty }{lim sup}\frac{\bar{F}\left(xy\right)}{\bar{F}\left(x\right)}=\lim_{y↓1}\underset{x\to +\infty }{lim inf}\frac{\bar{F}\left(xy\right)}{\bar{F}\left(x\right)}=1,$$

where $\bar{F}\left(x\right)$ is the tail probability with $\bar{F}\left(x\right)=1-F\left(x\right)$. The class C is a wide class of distributions commonly used in actuarial science, including the well-known Pareto, Burr, and loggamma distributions. Ng et al. [42] established a very useful result for the distributions of class C, which is given in the following lemma.

**Lemma** **3.** *Suppose that $\left\{Y_{j},j=1,2,\cdots \right\}$ is a sequence of i.i.d. non-negative random variables with common distribution function $F_{Y}\left(y\right)\in C$ and E(Y)<+∞. Taking $Q_{t}=\sum_{j=1}^{t}Y_{j}$, for any fixed γ>0, it holds uniformly for all y>γt that*

(33) $$P\left(Q_{t}-tE\left(Y\right)>y\right)∼t{\bar{F}}_{Y}\left(y\right),\;\;t\to +\infty ,$$

*in which the uniformity is understood in the following sense:*

$$\lim_{t\to +\infty }\underset{y\geq \gamma t}{\sup}\left|\frac{P(Q_{t}-tE\left(Y\right)>y)}{t{\bar{F}}_{Y}\left(y\right)}-1\right|=0.$$

Analogous to the infinite-time ruin probability ψ(u), for any fixed t=1,2,⋯, we define the finite-time ruin probability ψ(u,t) of the discrete-time risk model (2) as

$$\begin{array}{c} \psi \left(u,t\right)=P\left(T\leq t|U_{0}=u\right). \end{array}$$

In order to further study the asymptotic formula of ψ(u,t), which is also a core actuarial quantity, we revise Lemma 3 as follows.

**Lemma** **4.** *Suppose that $\left\{Y_{j},j=1,2,\cdots \right\}$ is a sequence of i.i.d. non-negative random variables with the common distribution function $F_{Y}\left(y\right)\in C$ and E(Y)<+∞. Define $Q_{t}=\sum_{j=1}^{t}Y_{j}$; then, for any fixed γ>0 and δ>0, it holds uniformly for all $y>\gamma t^{1+\delta }$ that*

(34) $$P\left(Q_{t}>y\right)∼t{\bar{F}}_{Y}\left(y\right),\;\;t\to +\infty .$$

**Proof.**  By the definition of class C, it follows for any fixed θ>0 and sufficiently large *y* that

$$\frac{{\bar{F}}_{Y}\left(\left(1+\theta \right)y\right)}{{\bar{F}}_{Y}\left(y\right)}\leq \frac{{\bar{F}}_{Y}\left(y+o\left(y\right)\right)}{{\bar{F}}_{Y}\left(y\right)}\leq \frac{{\bar{F}}_{Y}\left(\left(1-\theta \right)y\right)}{{\bar{F}}_{Y}\left(y\right)},$$

from which we can obtain

$$\begin{array}{cc} 1=\lim_{\theta ↓0}\underset{y\to +\infty }{lim inf}\frac{{\bar{F}}_{Y}\left(\left(1+\theta \right)y\right)}{{\bar{F}}_{Y}\left(y\right)}\; & \leq \underset{y\to +\infty }{lim inf}\frac{{\bar{F}}_{Y}\left(y+o\left(y\right)\right)}{{\bar{F}}_{Y}\left(y\right)}\\ \; & \leq \underset{y\to +\infty }{lim sup}\frac{{\bar{F}}_{Y}\left(y+o\left(y\right)\right)}{{\bar{F}}_{Y}\left(y\right)}\leq \lim_{\theta ↓0}\underset{y\to +\infty }{lim sup}\frac{{\bar{F}}_{Y}\left(\left(1-\theta \right)y\right)}{{\bar{F}}_{Y}\left(y\right)}=1. \end{array}$$

Hence, it holds that

(35) $$\lim_{y\to +\infty }\frac{{\bar{F}}_{Y}\left(y+o\left(y\right)\right)}{{\bar{F}}_{Y}\left(y\right)}=1.$$

Furthermore, by Lemma 3 and (35), it follows that

$$\begin{array}{cc} \lim_{t\to +\infty }\underset{y>\gamma t^{1+\delta }}{\sup}\left|\frac{P(Q_{t}>y)}{t{\bar{F}}_{Y}\left(y\right)}-1\right|\; & =\lim_{t\to +\infty }\underset{y>\gamma t^{1+\delta }}{\sup}\left|\frac{P(Q_{t}-tE\left(Y\right)>y-tE\left(Y\right))}{t{\bar{F}}_{Y}\left(y-tE\left(Y\right)\right)}\times \frac{{\bar{F}}_{Y}\left(y-tE\left(Y\right)\right)}{{\bar{F}}_{Y}\left(y\right)}-1\right|\\ \; & \leq \lim_{t\to +\infty }\underset{y>\gamma t^{1+\delta }}{\sup}\frac{{\bar{F}}_{Y}\left(y-tE\left(Y\right)\right)}{{\bar{F}}_{Y}\left(y\right)}\times \left|\frac{P(Q_{t}-tE\left(Y\right)>y-tE\left(Y\right))}{t{\bar{F}}_{Y}\left(y-tE\left(Y\right)\right)}-1\right|\\ \; & \;\;\;\;+\lim_{t\to +\infty }\underset{y>\gamma t^{1+\delta }}{\sup}\left|\frac{{\bar{F}}_{Y}\left(y-tE\left(Y\right)\right)}{{\bar{F}}_{Y}\left(y\right)}-1\right|\\ \; & =\lim_{y\to +\infty }\frac{{\bar{F}}_{Y}\left(y+o\left(y\right)\right)}{{\bar{F}}_{Y}\left(y\right)}\times \lim_{t\to +\infty }\underset{y>\gamma t^{1+\delta }}{\sup}\left|\frac{P(Q_{t}-tE\left(Y\right)>y-tE\left(Y\right))}{t{\bar{F}}_{Y}\left(y-tE\left(Y\right)\right)}-1\right|\\ \; & \;\;\;\;+\lim_{y\to +\infty }\left|\frac{{\bar{F}}_{Y}\left(y+o\left(y\right)\right)}{{\bar{F}}_{Y}\left(y\right)}-1\right|\\ \; & =0. \end{array}$$

The proof is then completed. □

Now, we give the precise large deviations of the aggregate claims, $S_{t}$, which is described in model (11).

**Theorem** **3.** *For our proposed model (2), let $F_{Y}\left(y\right)$ and E(Y) be the common distribution function and expectation of the claim sizes, respectively. Assuming $F_{Y}\left(y\right)\in C$ and E(Y)<+∞, then for any fixed γ>0 and δ>0, it holds uniformly for all $y>\gamma t^{1+\delta }$ that*

(36) $$P\left(S_{t}>y\right)∼\lambda_{2}\left(1+\beta \right)t{\bar{F}}_{Y}\left(y\right),\;\;t\to +\infty .$$

**Proof.**  Let $\left\{Y_{j},j=1,2,\cdots \right\}$ be a sequence of i.i.d. non-negative random variables, with their common distribution function denoted by $F_{Y}\left(y\right)$. Suppose that $\varphi_{S_{t}}\left(r\right)$ is the characteristic function of $S_{t}=\sum_{i=1}^{t}\sum_{j=1}^{N_{i}}Y_{i,j}$. With the same method to derive (12) and (13), we can obtain

(37) $$\varphi_{S_{t}}\left(r\right)=E\left[{\left(\varphi_{Y}\left(r\right)\right)}^{N_{1}+\cdots +N_{t}}\right],$$

where $\varphi_{Y}\left(r\right)$ is the characteristic function of *Y*.

On the other hand, direct calculation leads to

(38) $$\begin{array}{cc} E\left[\exp\left\{ir\sum_{j=1}^{N_{1}+\cdots +N_{t}}Y_{j}\right\}\right]\; & =\sum_{n}E\left[\exp\left\{ir\sum_{j=1}^{n}Y_{j}\right\}\times I_{\left\{N_{1}+\cdots +N_{t}=n\right\}}\right]\\ \; & =\sum_{n}{\left[E\exp\left\{irY\right\}\right]}^{n}\times P\left\{N_{1}+N_{2}+\cdots +N_{t}=n\right\}\\ \; & =E[{\left(\varphi_{Y}\left(r\right)\right)}^{N_{1}+\cdots N_{t}}]. \end{array}$$

We conclude after checking (37) and (38) that

(39) $$S_{t}\overset{d}{=}\sum_{j=1}^{N_{1}+\cdots +N_{t}}Y_{j},$$

where ”$\overset{d}{=}$” means the identical distribution.

For any $0<\eta <\lambda_{2}\left(1+\beta \right)$, we have

(40) $$\begin{array}{cc} \; & P\left(\sum_{j=1}^{N_{1}+\cdots +N_{t}}Y_{j}>y\right)\\ = & P\left(\sum_{i=1}^{t}N_{i}<\left\lfloor \left(\lambda_{2}\left(1+\beta \right)+\eta \right)t\right\rfloor ,\sum_{j=1}^{N_{1}+\cdots +N_{t}}Y_{j}>y\right)+P\left(\sum_{i=1}^{t}N_{i}\geq \left\lfloor \left(\lambda_{2}\left(1+\beta \right)+\eta \right)t\right\rfloor ,\sum_{j=1}^{N_{1}+\cdots +N_{t}}Y_{j}>y\right)\\ \leq & P\left(\sum_{j=1}^{\left\lfloor \left(\lambda_{2}\left(1+\beta \right)+\eta \right)t\right\rfloor }Y_{j}>y\right)+P\left(\sum_{i=1}^{t}N_{i}\geq \left\lfloor \left(\lambda_{2}\left(1+\beta \right)+\eta \right)t\right\rfloor \right)\\ = & \Delta_{1}+\Delta_{2}, \end{array}$$

in which ⌊·⌋ denotes the maximum integer not exceeding ${}^{\prime \prime }\cdot^{\prime \prime }$.

From Lemma 4, we know it holds uniformly for all $y>\gamma t^{1+\delta }$ that

(41) $$\Delta_{1}∼\left\lfloor \left(\lambda_{2}\left(1+\beta \right)+\eta \right)t\right\rfloor {\bar{F}}_{Y}\left(y\right).$$

As for $\Delta_{2}$, for t=1,2,⋯, we write

$$\sum_{i=1}^{t}N_{i}=\sum_{i=1}^{\left\lfloor t/2\right\rfloor }N_{2i}+\sum_{i=1}^{\lfloor t/2\rfloor +p}N_{2i-1},$$

where p=0 if *t* is a even number, and p=1 if *t* is an odd number. From the definition, we know that $\left\{N_{i},i=1,2,\cdots \right\}$ is a one-dependent stationary sequence with the common Poisson distribution of mean $\lambda_{2}\left(1+\beta \right)$ and m.g.f $M_{N}\left(r\right)=\exp\left\{\lambda_{2}\left(1+\beta \right)\left(e^{r}-1\right)\right\}$; then, it is easy to see that $\left\{N_{2i},1\leq i\leq \left\lfloor t/2\right\rfloor \right\}$ and $\left\{N_{2i-1},1\leq i\leq \left\lfloor t/2\right\rfloor +p\right\}$ are two sequences of i.i.d. random variables. Let $a=\lambda_{2}\left(1+\beta \right)+\eta$; by Cramér Theorem (Theorem 2.2.3 in Dembo and Zeitonui [43]), we have

(42) $$\begin{array}{cc} \Delta_{2}\; & =P\left(\sum_{i=1}^{\left\lfloor t/2\right\rfloor }N_{2i}+\sum_{i=1}^{\lfloor t/2\rfloor +p}N_{2i-1}\geq \left\lfloor \left(\lambda_{2}\left(1+\beta \right)+\eta \right)t\right\rfloor \right)\\ \; & \leq P\left(\sum_{i=1}^{\left\lfloor t/2\right\rfloor }N_{2i}\geq \left\lfloor \lambda_{2}\left(1+\beta \right)+\eta \right\rfloor \left\lfloor t/2\right\rfloor \right)+P\left(\sum_{i=1}^{\lfloor t/2\rfloor +p}N_{2i-1}\geq \left\lfloor \lambda_{2}\left(1+\beta \right)+\eta \right\rfloor \left(\left\lfloor t/2\right\rfloor +p\right)\right)\\ \; & ∼e^{-\lfloor t/2\rfloor I(a)}+e^{-(\lfloor t/2\rfloor +p)I(a)}\to 0,\;\;t\to +\infty , \end{array}$$

in which $I\left(a\right)=\underset{-\infty <r<+\infty }{\sup}\left\{ar-\log\left(M_{N}\left(r\right)\right)\right\}>0$.

Combining (40)–(42) gives

(43) $$P\left(\sum_{j=1}^{N_{1}+\cdots +N_{t}}Y_{j}>y\right)≲\left\lfloor \left(\lambda_{2}\left(1+\beta \right)+\eta \right)t\right\rfloor {\bar{F}}_{Y}\left(y\right).$$

On the other hand, it holds that

(44) $$\begin{array}{cc} P\left(\sum_{j=1}^{N_{1}+\cdots +N_{t}}Y_{j}>y\right)\; & \geq P\left(\sum_{i=1}^{t}N_{i}\geq \left\lfloor \left(\lambda_{2}\left(1+\beta \right)-\eta \right)t\right\rfloor ,\sum_{j=1}^{N_{1}+\cdots +N_{t}}Y_{j}>y\right)\\ \; & \geq P\left(\sum_{i=1}^{t}N_{i}\geq \left\lfloor \left(\lambda_{2}\left(1+\beta \right)-\eta \right)t\right\rfloor ,\sum_{j=1}^{\left\lfloor \left(\lambda_{2}\left(1+\beta \right)-\eta \right)t\right\rfloor }Y_{j}>y\right)\\ \; & =P\left(\sum_{i=1}^{t}N_{i}\geq \left\lfloor \left(\lambda_{2}\left(1+\beta \right)-\eta \right)t\right\rfloor \right)\times P\left(\sum_{j=1}^{\left\lfloor \left(\lambda_{2}\left(1+\beta \right)-\eta \right)t\right\rfloor }Y_{j}>y\right), \end{array}$$

in which

(45) $$\begin{array}{cc} P\left(\sum_{i=1}^{t}N_{i}\geq \left\lfloor \left(\lambda_{2}\left(1+\beta \right)-\eta \right)t\right\rfloor \right)\; & =P\left(\sum_{i=1}^{t}N_{i}-\lambda_{2}\left(1+\beta \right)t\geq \left\lfloor \left(\lambda_{2}\left(1+\beta \right)-\eta \right)t\right\rfloor -\lambda_{2}\left(1+\beta \right)t\right)\\ \; & \geq P\left(\sum_{i=1}^{t}N_{i}-\lambda_{2}\left(1+\beta \right)\geq -\eta t\right)\\ \; & \geq P\left(\left|\sum_{i=1}^{t}N_{i}-\lambda_{2}\left(1+\beta \right)t\right|\leq \eta t\right)\to 1,\;\;t\to +\infty , \end{array}$$

because of the fact that

$$\begin{array}{cc} \; & P\left(\left|\sum_{i=1}^{t}N_{i}-\lambda_{2}\left(1+\beta \right)t\right|>\eta t\right)\\ \leq & P\left(\left|\sum_{i=1}^{\left\lfloor t/2\right\rfloor }\left[N_{2i}-\lambda_{2}\left(1+\beta \right)\right]\right|>\eta \left\lfloor t/2\right\rfloor \right)+P\left(\left|\sum_{i=1}^{\lfloor t/2\rfloor +p}\left[N_{2i-1}-\lambda_{2}\left(1+\beta \right)\right]\right|>\eta \left(\left\lfloor t/2\right\rfloor +p\right)\right)\\ \to & 0,\;\;t\to +\infty , \end{array}$$

obtained from the classical law of large numbers.

Then, combining (44), (45), and Lemma 4 yields

(46) $$P\left(\sum_{j=1}^{N_{1}+\cdots +N_{y}}Y_{j}>y\right)≳\left\lfloor \left(\lambda_{2}\left(1+\beta \right)-\eta \right)t\right\rfloor {\bar{F}}_{Y}\left(y\right).$$

Generally, letting η↓0 in (43) and (46) and keeping (39) in mind, we finally conclude that

$$P\left(S_{t}>y\right)=P\left(\sum_{j=1}^{N_{1}+\cdots +N_{t}}Y_{j}>y\right)∼\left\lfloor \lambda_{2}\left(1+\beta \right)t\right\rfloor {\bar{F}}_{Y}\left(y\right)∼\lambda_{2}\left(1+\beta \right)t{\bar{F}}_{Y}\left(y\right).$$

Then, the proof is completed. □

With the help of the above conclusion, we can manifest the asymptotic formula for the finite-time ruin probability in the following theorem.

**Theorem** **4.** *Under the conditions of Theorem 3, for any fixed γ>0 and δ>0, the asymptotic formula*

(47) $$\psi \left(u,t\right)∼\lambda_{2}\left(1+\beta \right)t{\bar{F}}_{Y}\left(u\right)$$

*holds uniformly for all $u>\gamma t^{1+\delta }$ as t→+∞.*

**Proof.**  From the definition of finite-time ruin probability, it is clear that

(48) $$\begin{array}{cc} \psi (u,t)\; & =P\left(\underset{m\in \{0,1,\cdots ,t\}}{\sup}\left(S_{m}-W_{m}\right)>u\right)\\ \; & \geq P\left(S_{t}-W_{t}>u\right)\\ \; & =P\left(S_{t}>u+W_{t}\right)\\ \; & =P\left(S_{t}>u+\frac{\lambda_{1}}{1-\alpha }tE\left(X\right)+W_{t}-\frac{\lambda_{1}}{1-\alpha }tE\left(X\right)\right). \end{array}$$

Noting that $W_{t}=\sum_{i=1}^{t}P_{i}=\sum_{i=1}^{t}\sum_{k=1}^{M_{i}}X_{i,k}$ and keeping (9) in mind, for any η>0, we have

$$\begin{array}{cc} P\left(\left|W_{t}-\frac{\lambda_{1}}{1-\alpha }tE\left(X\right)\right|\geq \eta t\right)\; & =P\left(\left|\frac{1}{t}\sum_{i=1}^{t}P_{i}-\frac{\lambda_{1}}{1-\alpha }E\left(X\right)\right|\geq \eta \right)\\ \; & \leq \frac{E{\left(\frac{1}{t}\sum_{i=1}^{t}P_{i}-\frac{\lambda_{1}}{1-\alpha }E\left(X\right)\right)}^{2}}{\eta^{2}}\\ \; & =\frac{1}{{\left(\eta t\right)}^{2}}\sum_{i,j=1}^{t}Cov\left(P_{i},P_{j}\right)\\ \; & =\frac{\lambda_{1}E\left(X^{2}\right)}{\left(1-\alpha \right){\left(\eta t\right)}^{2}}\left(t+2\left(t-1\right)\alpha +2\left(t-2\right)\alpha^{2}+\cdots +2\alpha^{t-1}\right)\\ \; & \leq \frac{2\lambda_{1}E\left(X^{2}\right)\left(1-\alpha^{t}\right)}{t\eta^{2}{\left(1-\alpha \right)}^{2}}\to 0,\;\;t\to +\infty , \end{array}$$

from which we can obtain

$$\lim_{t\to +\infty }\underset{u>\gamma t^{1+\delta }}{\sup}\frac{1}{u}\left(\frac{\lambda_{1}}{1-\alpha }tE\left(X\right)+W_{t}-\frac{\lambda_{1}}{1-\alpha }tE\left(X\right)\right)=0.$$

Then, for any θ>0, if *t* is sufficiently large such that *u* is sufficiently large, it holds that

$$P\left(S_{t}>u+\theta u\right)\leq P\left(S_{t}>u+\frac{\lambda_{1}}{1-\alpha }tE\left(X\right)+W_{t}-\frac{\lambda_{1}}{1-\alpha }tE\left(X\right)\right)\leq P\left(S_{t}>u-\theta u\right),$$

Furthermore, by Theorem 3 and let θ↓0, we have

(49) $$P\left(S_{t}>u+\frac{\lambda_{1}}{1-\alpha }tE\left(X\right)+W_{t}-\frac{\lambda_{1}}{1-\alpha }tE\left(X\right)\right)∼\lambda_{2}\left(1+\beta \right)F_{Y}\left(u\right),\;\;\mathrm{uniformly}\;\mathrm{for}\;u>\gamma t^{1+\delta }\;\mathrm{as}\;t\to +\infty .$$

Plugging (49) into (48) gives

(50) $$\psi \left(u,t\right)≳\lambda_{2}\left(1+\beta \right)t{\bar{F}}_{Y}\left(u\right).$$

On the other hand, for any fixed γ>0 and δ>0, we have uniformly for all $u>\gamma t^{1+\delta }$ that

$$\psi \left(u,t\right)=P\left(\underset{m\in \{0,1,\cdots ,t\}}{\sup}\left(S_{m}-W_{m}\right)>u\right)\leq P\left(S_{t}>u\right)∼\lambda_{2}\left(1+\beta \right)t{\bar{F}}_{Y}\left(u\right).$$

which implies

(51) $$\psi \left(u,t\right)≲\lambda_{2}\left(1+\beta \right)t{\bar{F}}_{Y}\left(u\right).$$

Therefore, we complete the proof by combining (50) and (51). □

**Remark** **9.** *Applying Lemma 3 instead of Lemma 4 in Theorem 3, it is not difficult to see that the precise large deviation (36) also holds uniformly for all y>γt. In this paper, we restrict ourselves to the interval $y>\gamma t^{1+\delta }$ in order to provide convenience for investigating the finite-time ruin probability ψ(u,t). Moreover, we can prove that the asymptotic formula (47) in Theorem 4 holds uniformly for all u∈Ω={u;t=o(u)}, which includes $u>\gamma t^{1+\delta }$ as a special case. In practice, when t is large enough, we can asymptotically estimate ψ(u,t) by $\lambda_{2}\left(1+\beta \right)t{\bar{F}}_{Y}\left(u\right)$, as the size of claims belong to the distributions of class C and the insurer’s initial surplus is adequate in the sense of $u>\gamma t^{1+\delta }$.*

## 6. Numerical Examples

In this section, we aim to perform some numerical simulations to demonstrate and assess the Lundberg adjustment coefficient and the Lundberg approximation results for the infinite-time ruin probability ψ(u), as well as the asymptotic formula for the finite-time ruin probability ψ(u,t), of our proposed model.

**Example** **1.** *We suppose that the gain amount X and the claim size Y follow exponential distributions that have means $1/\mu_{1}$ and $1/\mu_{2}$, respectively. Therefore, we have the moment generating functions of X and Y as follows:*

(52) $$M_{X}\left(-r\right)=\frac{\mu_{1}}{\mu_{1}+r},\;\;M_{Y}\left(r\right)=\frac{\mu_{2}}{\mu_{2}-r},\;\;r>0.$$

*Then, from Theorem 2, c(r)=0 is equivalent to*

(53) $$\begin{array}{c} \lambda_{1}\frac{1}{\left(1-\alpha \right)\mu_{1}+r}=\lambda_{2}\frac{\left(1+\beta \right)\mu_{2}-r}{{\left(\mu_{2}-2\right)}^{2}}. \end{array}$$

*The unique positive solution to Equation (53) can be found but appears tedious. In what follows, we give some numerical results to show the properties and performance of R and $e^{-Ru}$.*

*Without loss of generality, we set $\lambda_{1}=1$, $\lambda_{2}=0.4$, $\mu_{1}=1$, and $\mu_{2}=0.5$, and then, calculate and discuss the Lundberg adjustment coefficient R and the approximated ruin probability $e^{-Ru}$ for different values of α and β. When we consider the impacts of α and β on the main results, it should be noted that the relative safety loading condition (26) has to be satisfied, i.e.,*

(54) $$\begin{array}{c} \frac{\lambda_{1}}{1-\alpha }\cdot \frac{1}{\mu_{1}}>\lambda_{2}\cdot \left(1+\beta \right)\cdot \frac{1}{\mu_{2}}, \end{array}$$

*which, in our parameter scenario, implies*

(55) $$\begin{array}{c} \frac{1}{1-\alpha }>0.8\left(1+\beta \right). \end{array}$$

**Figure 1:**
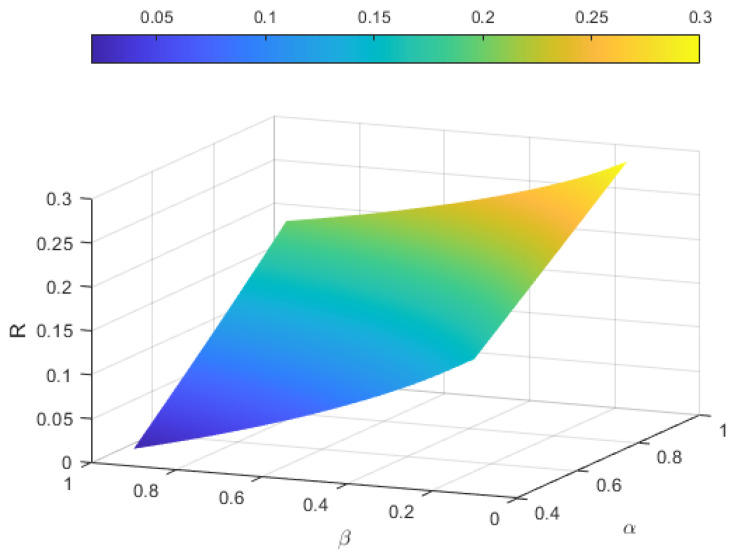
Lundberg adjustment coefficients corresponding to different α and β.

*Table 1 gives the computed values of Lundberg adjustment coefficients corresponding to different values of α and β. We also illustrate these results in Figure 1, from which it can be clearly seen that R increases as α increases, implying that the insurance portfolio would become less and less dangerous because the approximated infinite-time ruin probability $e^{-Ru}$ decreases. In the same sense, when β increases, R will decrease, meaning that there could be higher risks in the insurance portfolio. (In Table 1, the notation ″−″ means that the values of α and β do not satisfy the relative safety loading condition, and the Lundberg adjustment coefficients are not considered for these situations.)*

*In order to evaluate the performance of the approximated infinite-time ruin probability $e^{-Ru}$, we fix α=β=0.5 in the proposed risk model and compute the true ruin probabilities corresponding to different values of u by the Monte Carlo method used in Albrecher and Kantor [44]. For this purpose, we randomly draw sample paths according to the Poisson INAR(1) process and the Poisson INMA(1) process for the premium arrivals $\left\{M_{t},t=1,2,\cdots \right\}$ and the claim numbers $\left\{N_{t},t=1,2,\cdots \right\}$, respectively. Afterwards, we simulate the surplus process (2) starting at $U_{0}=u$, with the premium amounts and claim sizes following the given exponential distributions. These simulations are replicated n=3000 times; then, the trajectories with negative values (i.e., ruin event occurs) are counted, and we denote this number by $n_{1}$. Hence, the infinite-time ruin probability ψ(u) can be estimated by*

(56) $$\begin{array}{c} \hat{\psi }\left(u\right)=\frac{n_{1}}{n}. \end{array}$$

*In addition, because of the fact that $U_{t}\to +\infty$ with probability one as t→+∞ when the relative safety loading condition holds, we know that $U_{t}$ will never become negative when t is large enough. Therefore, it is necessary for us to choose a suitable $T_{\mathrm{st}}$ at which we should stop the simulated surplus process for each sample path if the ruin event does not occur before this time. As a consequence, (56) is actually the estimate of the finite-time ruin probability $\psi \left(u,T_{\mathrm{st}}\right)=P\left(T\leq T_{\mathrm{st}}|U_{0}=u\right)$. In this paper, we set $T_{\mathrm{st}}=1000$. In practice, we can choose larger values for $T_{\mathrm{st}}$ so that the bias of the estimator for ψ(u) is less significant.*

**Figure 2:**
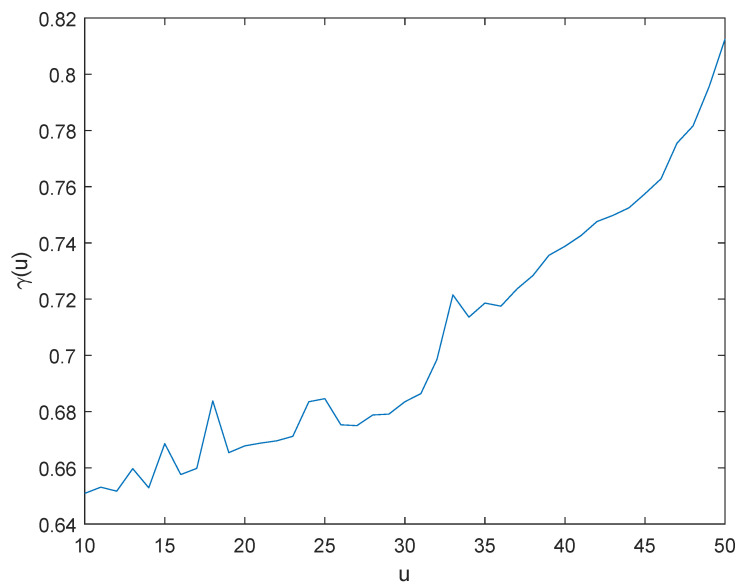
The simulated and approximated ruin probabilities with respect to different values of *u*.

*In Table 2 and Figure 2, we compare the simulated ruin probability with the approximated ruin probability. As can be seen, when u grows, both of the ruin probabilities approach zero. However, as alternatives to the true ruin probabilities, the approximations do not work well when the values of u are small. We can explain these results with the following three reasons. Firstly, as the limit of ψ(u) as u→+∞, $e^{-Ru}$ may be very different than ψ(u) at the beginning. Secondly, the simulated infinite-time ruin probabilities are indeed the estimated values for the finite-time ruin probability $\psi (u,T_{\mathrm{st}})$, which are smaller than the true values of ψ(u). Thirdly, the total number of simulated trajectories n and the chosen time $T_{\mathrm{st}}$ affect the simulated results. We could increase n and $T_{\mathrm{st}}$ to improve the performance, but a longer run time is needed.*

**Figure 3:**
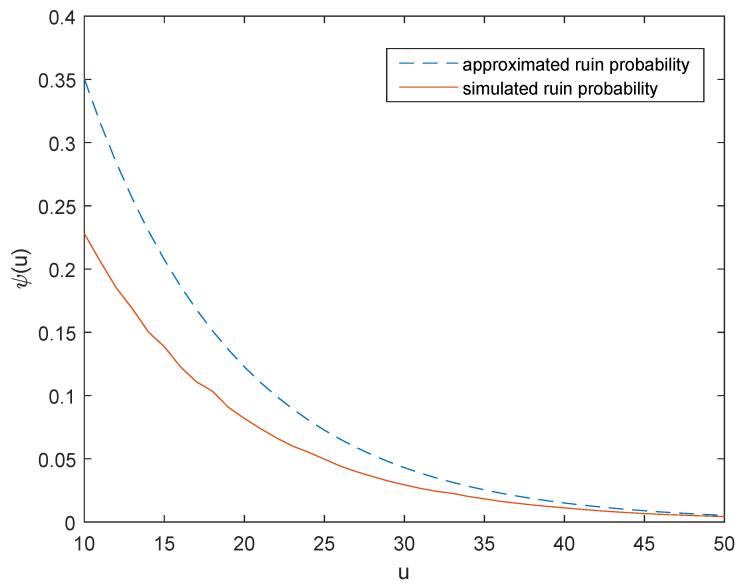
The values of ratio γ(u) with respect to different *u*.

*On the other hand, the values of simulated ruin probability and the approximated ruin probability become closer and closer with the increase in u, implying that the approximation method could work better as u grows. To strengthen this statement, we define $\gamma \left(u\right)=\frac{\hat{\psi }\left(u\right)}{e^{-Ru}}$, and then, calculate the values of γ(u) with respect to different values of u; the results are listed in the last column of Table 2. It can be seen that γ(u) approaches 1 asymptotically, which indicates that it is valid to take $e^{-Ru}$ as the approximated result for $\hat{\psi }\left(u\right)$ and, furthermore, for ψ(u) when u is large enough. Figure 3 also illustrates this conclusion visually. In practice, an insurer is always required to hold a huge number of initial surplus to guarantee its solvency under certain regulatory frameworks; therefore, the approximation method is of importance and is applicable in the risk management of insurance.*

**Example** **2.** *We suppose that the gain amount X is distributed by the exponential distribution with mean of $1/\mu_{1}$, and the claim size Y follows the Pareto distribution, which has shape parameter $\tau_{1}$ and scale parameter $\tau_{2}$, i.e., the distribution function $F_{Y}\left(y\right)$ is given by $F_{Y}\left(y\right)=1-{\left(\frac{\tau_{2}}{\tau_{2}+x}\right)}^{\tau_{1}},\;\;y>0$. To perform the calculations, we set $\lambda_{1}=1$, $\mu_{1}=1$, $\lambda_{2}=0.1$, $\tau_{1}=3$, $\tau_{2}=16$, and α=β=0.5. It is not difficult to check that these values satisfy the relative safety loading condition (26). Our goal is to compare the asymptotic result $\lambda_{2}\left(1+\beta \right)tF_{Y}\left(y\right)$ (AS for simplification) with the simulated results of the finite-time ruin probabilities obtained using the Monte Carlo method (MC for simplification). As can be seen from Table 3, the ratio of MC to AS becomes closer and closer to one as t increases for different u, indicating that the asymptotic formula stated in Theorem 4 is valid and applicable in practice.*

## 7. Conclusions

In this paper, we examine a generalization of the classical discrete-time risk model of an insurance portfolio with stochastic premiums, using a Poisson INAR(1) process and a Poisson INMA(1) process to fit the temporal dependence among the premium numbers and the temporal dependence among the claim numbers, respectively. We give the explicit expression for the function satisfied by the Lundberg adjustment coefficient and find the Lundberg approximation formula for the infinite-time ruin probability. Furthermore, we discuss and analyze the impact of the two thinning parameters and manifest that the dependence structure in the model has a significant influence on the risk of the surplus process in an insurance company. When the claim sizes follow a class of heavy-tailed distributions, we establish the large deviations of the aggregate claims and investigate the asymptotic formula for the finite-time ruin probability. In the numerical examples, we use MATLAB to randomly draw the sample paths of the proposed surplus process and compute estimates of the true ruin probabilities corresponding to different values of *u* using the Monte Carlo method. From the simulated results, it can be seen that the approximation formula and asymptotic formula we obtained are effective. Furthermore, these two formulas are much simpler to use for calculating and estimating the ruin probabilities than the Monte Carlo method.

As for future work, we could implement the same methodology by applying the time series for count data with other distributed innovations or an arbitrary innovations’ distribution. Generally, using the same approach as that in Lemma 1 and Lemma 2, we can extend (14) and (20) to

$$P_{M_{1}+\cdots +M_{t}}\left(s\right)=P_{M_{1}}\left(h_{t}\left(s\right)\right)\prod_{i=1}^{t-1}P_{\varepsilon }\left(h_{i}\left(s\right)\right),$$

and

$$P_{N_{1}+\cdots +N_{t}}\left(s\right)=P_{\eta }\left(s\right)P_{\eta }\left(\beta s+\left(1-\beta \right)\right){\left[P_{\eta }\left(\beta s^{2}+\left(1-\beta \right)s\right)\right]}^{t-1},$$

respectively. Therefore, if we could derive the explicit expression of c(r), the properties of the solution to the equation c(r)=0 can be discussed, and the adjustment coefficient can be obtained to measure the risk.

Additionally, we could adopt some higher-order processes to make the insurance risk model much more practical and flexible. In this situation, it becomes more challenging to find the expressions of $P_{M_{1}+\cdots +M_{t}}\left(s\right)$ and $P_{N_{1}+\cdots +N_{t}}\left(s\right)$. As a consequence, there might be some difficulties in deriving c(r) and defining the adjustment coefficient for an insurance portfolio.

On the other hand, instead of fixing the distributions and the parameters to illustrate the results by simulation, we can use the real dataset to fit the distributions and obtain the statistical estimates of the parameters, so that the ruin problems of the risk model could be analyzed in a more scientific way. 

## Figures and Tables

**Table 1:** Lundberg adjustment coefficients for different α and β.

	β								
α	0.1	0.2	0.3	0.4	0.5	0.6	0.7	0.8	0.9
0.1	0.0680	0.0414	0.0183	-	-	-	-	-	-
0.2	0.0968	0.0706	0.0481	0.0282	0.0104	-	-	-	-
0.3	0.1256	0.1000	0.0781	0.0588	0.0416	0.0259	0.0115	-	-
0.4	0.1545	0.1295	0.1082	0.0897	0.0731	0.0581	0.0443	0.0316	0.0198
0.5	0.1834	0.1591	0.1386	0.1208	0.1049	0.0906	0.0776	0.0655	0.0544
0.6	0.2124	0.1888	0.1691	0.1522	0.1371	0.1236	0.1113	0.1000	0.0895
0.7	0.2415	0.2187	0.2000	0.1839	0.1698	0.1571	0.1457	0.1351	0.1254
0.8	0.2707	0.2489	0.2312	0.2162	0.2031	0.1913	0.1807	0.1711	0.1622
0.9	0.3000	0.2794	0.2630	0.2491	0.2370	0.2264	0.2167	0.2080	0.2000

**Table 2:** Comparison of the simulated and approximated ruin probability.

*u*	$\hat{\psi }\left(u\right)$	$e^{-\mathrm{Ru}}$	$\gamma \left(u\right)=\frac{\hat{\psi }\left(u\right)}{e^{-\mathrm{Ru}}}$
10	0.2280	0.3503	0.6509
15	0.1386	0.2073	0.6686
20	0.0819	0.1227	0.6678
25	0.0497	0.0726	0.6846
30	0.0294	0.0430	0.6835
35	0.0183	0.0254	0.7186
40	0.0112	0.0151	0.7388
45	0.0067	0.0089	0.7575
50	0.0043	0.0053	0.8125

**Table 3:** Comparison of the simulated results and the asymptotic results for ψ(u,t).

		u=60	u=70	u=80	u=90	u=100
	AS	0.0760	0.0560	0.0437	0.0300	0.0210
t=50	MC	0.0700	0.0483	0.0347	0.0258	0.0197
	AS/MC	1.0860	1.1595	1.2576	1.1628	1.0660
	AS	0.0703	0.0487	0.0440	0.0280	0.0200
t=40	MC	0.0560	0.0386	0.0278	0.0206	0.0157
	AS/MC	1.2563	1.2596	1.5840	1.3592	1.2739
	AS	0.0517	0.0360	0.0330	0.0170	0.0140
t=30	MC	0.0420	0.0290	0.0208	0.0155	0.0118
	AS/MC	1.2305	1.2423	1.5840	1.0985	1.1856
	AS	0.0377	0.0247	0.0223	0.0140	0.0120
t=20	MC	0.0280	0.0193	0.0139	0.0103	0.0079
	AS/MC	1.3456	1.2768	1.6043	1.3570	1.5190
	AS	0.0190	0.0130	0.0113	0.0077	0.0063
t=10	MC	0.0140	0.0097	0.0069	0.0052	0.0039
	AS/MC	1.3575	1.3402	1.6377	1.4862	1.6090

## Data Availability

Not applicable.

## Appendix A

In this appendix, we explicate the motivation and rationale of our proposed model (2) by the following descriptions, in order to make this paper more accessible to the readers. Let us begin with the classical discrete-time Lundberg–Cramér risk model

(A1) $$U_{t}=U_{t-1}+c-L_{t},\;t=1,2,\cdots ,$$

where $U_{t}$ corresponds to the surplus of an insurance portfolio at time *t*, with $U_{0}=u$ being the initial surplus; *c* being the constant premium income per period, and $L_{t}$ representing the aggregate claim amount in period *t* that is defined as

(A2) $$L_{t}=\sum_{j=1}^{N_{t}}Y_{t,j},$$

in which $N_{t}$ denotes the number of claims and $Y_{t,j}$ is the size of the *j*th payment to the insured in period *t*. After recursively calculating, it is easy to see that the risk model (A1) can be rewritten as

$$U_{t}=u+ct-\sum_{i=1}^{t}L_{i}=u+ct-\sum_{i=1}^{t}\sum_{j=1}^{N_{i}}Y_{i,j},\;t=1,2,\cdots ,$$

which is equivalent to the model (1) by denoting $N_{t}^{0}=\sum_{i=1}^{t}N_{i}$.

For simplicity, it is assumed that the claim numbers of different periods are independent in the Lundberg–Cramér risk model, i.e., the claim number process $\left\{N_{t},t=1,2,\cdots \right\}$ is a sequence of i.i.d. random variables, which is certainly not realistic. As a consequence, ref. [11] proposes some new discrete-time risk models, where the Poisson INMA(1) process and Poisson INAR(1) process are used to describe the dependence structures among the numbers of claims. That is to say, the claim number process $\left\{N_{t},t=1,2,\cdots \right\}$ satisfies

$$N_{t}=\alpha \circ N_{t-1}+\varepsilon_{t},\;\;t=2,3,\cdots ,$$

or

$$N_{t}=\beta \circ \eta_{t-1}+\eta_{t},\;\;t=1,2,\cdots .$$

On the other hand, both of the above two types of risk models suppose that the premiums are collected with positive deterministic constant rate *c*, which is also lacks the ability of describing the real situation of insurance portfolio. As an alternative to this case, ref. [18] proposes the risk model with stochastic premiums that can be expressed as

(A3) $$U_{t}=U_{t-1}+P_{t}-L_{t},\;t=1,2,\cdots ,$$

where $L_{t}$ is defined by (A2), and $P_{t}$ aggregates the premiums in period *t* that is defined as

(A4) $$P_{t}=\sum_{k=1}^{M_{t}}X_{t,k},$$

in which $M_{t}$ counts the number of individual income, and $X_{t,k}$ represents the amount of the *k*th premium income for the insurance portfolio in period *t*.

In the risk model (A3), it should be noted that both the premium number process $\left\{M_{t},t=1,2,\cdots \right\}$ and the claim number process $\left\{N_{t},t=1,2,\cdots \right\}$ are supposed to be sequences of i.i.d. random variables. Therefore, the goal of this paper is to introduce the idea of [11] into the risk model with stochastic premiums by using time series for count random variables to fit the temporal dependence among $\left\{M_{t},t=1,2,\cdots \right\}$ and $\left\{N_{t},t=1,2,\cdots \right\}$, respectively. Furthermore, considering some insurance practices (please see Remarks 2 and 3), we assume that $\left\{M_{t},t=1,2,\cdots \right\}$ constitutes a Poisson INAR(1) process that satisfies

$$M_{t}=\alpha \circ M_{t-1}+\varepsilon_{t},\;\;t=2,3,\cdots ,$$

and $\left\{N_{t},t=1,2,\cdots \right\}$ constitutes a Poisson INMA(1) process that satisfies

$$N_{t}=\beta \circ \eta_{t-1}+\eta_{t},\;\;t=1,2,\cdots .$$

Our proposed risk model can also be generalized in several aspects to make itself more flexible and applicable, as discussed in the Conclusions.
